# Medical Musings

**Published:** 1899-04

**Authors:** 


### *MEDICAL MUSINGS.*

---

Doctors are human, and as such are fond of good jokes. We are indebted to *The Doctor's Factotum* for most of what follows.

---

In pleasant conversation they predicted their fine harvest in Cuba.

The following conversation, overheard some months ago, may be of interest at the present time:

A few friendly Bacteria were drinking coffee after having dined together at the Royal Intestine Club one evening, when Diphtheria Bacillus remarked to Typhoid Bacillus: "Typhy, old man, you are one of the lucky devils; business goes along well with you, while we fellows see our splendid old-established houses crumbling before the modern school. Now, there is poor old Smallpox. I have heard Amœbic Dysentery and his grandfather Cholera say they remember when Smallpox did nearly all the business on the continent."

"That's just it," said Measles, in a high-pitched, irritable voice; "you fellows get your business so extensive that you attract attention, and people want to know who you are and what you are and all the rest of it. Now, I am satisfied to work along quietly, unless I get into a new place, and then for a little while it pays to do a skyrocket business."

It was evident nobody liked Measles—he was a thin, red-faced little fellow, with a certain affectionate way, however, that one could see would take with children—everybody knew, moreover, that he was a good, quiet business man, who held his place third in the mortality statistics in the Eruptive Fever Union. It was this knowledge of his ability that gained him entrée into the best clubs. That day he and Chickenpox had strolled in, the latter having been put up at the club by his uncle, Smallpox, who, of course, belonged to all the clubs.

Over by the window, reading the Anti-Serum Gazette, sat Tubercle Bacillus. You could see that in his younger days he had been quite a fine looking fellow. He rose, and, approaching the group, said: "Well, boys, how is business?" "Pretty good," said two or three. "Elegant," said Measles, in his same piping, impudent voice.

"I see, Diphtheria, my boy," continued Tubercle Bacillus, not noticing Measles, "England is still with you. She will not accept Antitoxin; they are weakening, though, I am afraid. Now, I have made it a rule, when these investigations are too searching, to get out of the way for a little while by taking up business somewhere else, and you know we can fool these old doctors that way every time. Why, one day Staphylococcus, who is now in business with me (clever all-round fellow he is, too), came running into my office and said: 'Have you seen that Koch has definitely discovered you?' 'Yes, I saw that,' I replied quietly;' but don't worry. Aufrecht saw me some years ago, and even Conheim and Klebs came as near to it without finding me as any one.' And, boys, I say to you, don't mind being seen; you can sometimes fool them better when they see you, for then they work with you and don't upset your business with a great list of drugs. In my late unpleasantness with Koch over the tuberculin affair, I was sorely put to it, but one day I called Streptococcus and said that he and Staphylococcus must transfer the bulk of my business from the lungs to the peritoneum, and that I wanted my own men to be first put in charge of the new place of business, and that later Strepto and Staphylococcus and their men could take hold.

"Well, you would be surprised how nicely business is going on there. Before this transfer every little country practitioner could upset our lung business; to-day there are only a few big consultants ever disturb us in the peritoneum. I have been thinking seriously of giving up the lungs altogether."

"It's all right," said Typhoid, "for you and Diphtheria to talk of shifting your business. Now, I can do business in but one place, and they seem to be getting on to me there fast. While Pfeiffer and Grunbaum were working on poor old Cholera (whom I would have taken into business did he not meet with so many difficulties at port), I was so nervous, as we do business almost in the same street, that my mortality record in Baltimore, one of my best cities, was absurdly low. What was the result of that work? Why, now, with the improved methods, they can spot me almost every time; and to me that means breaking up my business and ultimately knocking me out."

It was evident that Typhoid was depressed and thought that his business looked gloomy; he, however, called the servant, Ptomaine, to take the orders for drinks. Tubercle and Typhoid called for milk punches, Typhoid adding that he wanted a dash of Woodbridge Antiseptic, which he found improved him so much. "Or," said he, "give me a bottle of Aerated Schuylkill Water. That always improves me."

"What you grumbling about, Typhoid?" said Yellow Fever. "You, who have no port restrictions, ought to be happy enough now that war is declared."

"You don't say so!" said Malaria; "that makes things look up all around."

Malaria was an Italian by birth; he had never been cultivated, however, and, being a foreigner, his methods of business were a little obscure, even to Typhoid, who at one time was supposed to be in partnership with him.

"I have it," said Typhoid. "Let us watch the medical appointments, and if they are what we want, Yellow and you, Malaria, and I will go to Cuba and see what we can do."

"Did you see," said Malaria, "that one of your friends, Yellow, is at the head of the Medical Department?"

"Not Sanarelli," said Yellow, anxiously. "No," replied Malaria, "he is an American."

"You don't mean Ster—what—that settles it," said Yellow, jubilantly.

"There is not a name on the list I ever heard of, and I'll bet," continued Malaria, laughingly, "not one of them has ever heard of me, much less seen me. But they all know about Quinine," he added, dolefully.

"Never mind," said Typhoid, "we can fool them together on the Temperature charts. I will write a private letter to Johns Hopkins Hospital and ask if they have any objection to Messrs.

Malaria and Typhoid doing a little business together for a few weeks."

Just then Ptomaine came in with the drinks.

"Ptomaine," said Typhoid, "do you want to go to Cuba with me as my servant? You are a smart fellow and can probably fool those doctors easier than I."

Ptomaïne was delighted, and agreed to join them the next day.

The party consisted of Yellow Fever, Typhoid Fever, Plasmodium Malariæ, and Ptomaine (servant).

The conversation herein related was overheard by Bichloride and his brother, Calcium Chlorinatus, and the young fellow Formaldehyde, also Quinine, who were doing detective duty in the interest of the United States Government and the army. When, however, the appointments to Cuba came, only Quinine was appointed, and the rest were kept at home to do hospital duty. "It is outrageous, for we (and I must here acknowledge that I am Bichloride) all agreed that night to get those fellows by the neck, and I firmly believe if our services had been accepted we would have saved a lot of lives. As it was, the services of the Competent Antiseptic and Disinfectant Union, of which we are members, were not accepted, so that I have since learned that Typhoid has opened new branches all over the United States, and expects to do the biggest business on record. Now, remember right here that we didn't advocate any special treatment. Our services were simply volunteered with the idea of disinfecting and keeping clean the utensils and camp. Typhoid, I hear, is actually planning to take some of the present army medical staff into partnership; for the honor of the profession I hope this is not true."—*N. Y. Times.*

---

### MUSIC HATH CHARMS, ETC.

A correspondent sends the following festive effusion: "It has been suggested that music might prove a useful adjunct (in some cases, at least) where the usual treatment by medicine had not proved satisfactory. I venture to suggest the following well known airs as being suitable for the cases enumerated, viz.:

Retarded labor from inertia ("Coming Through the Rye").

Chronic aphonia ("The Lost Chord").

Melancholia ("The Heart Bowed Down").

Epilepsy ("Let Me Like a Soldier Fall").

Cases of chronic deafness ("Come Back to Erin").

Pyrexia ("McCoolin").

Cases of doubtful diagnosis ("Oh, Dear, What Can the Matter Be?").—A. D., *Medical Press and Circular.*

---

### REQUIESCAT IN PACE.

No more he'll ever greet us,
He now is with the blest;
He got appendicitis,
And the doctors did the rest.—*Judge.*

---

### A CLOSE CALL.

Mrs. Sharp—See here, Robert! What are all those red, white and blue disks I found in your coat pocket?

Dr. S. (Professor of Histology, etc.)—Eh? Why, those are—that is, I use chi—I mean disks—to illustrate my lecture on the blood. You see, the white ones represent the white corpuscles, and the red ones the red corpuscles of the blood.

Mrs. S.—And what do the blue ones represent?

Dr. S.—The blue ones? Oh, yes—h'm—why, they represent the corpuscles of the venous blood.—*Funny-Bone.*

---

### FINANCIAL PHLEBOTOMY.

"I had supposed until yesterday, doctor, that the days of the bleeding of patients were past."

"And so they are. But what changed your mind?"

"The bill you sent me."—*Harper's Weekly.*

---

### A SENSIBLE PRECAUTION.

Fair Patient—Doctor, my memory has become very bad of late.

Doctor—Indeed. In these cases it is my invariable rule to ask for my fee in advance.

### PROGRESS OF SURGICAL SCIENCE.

Apropos of the craze for removal of the stomach, *Atlantic Medical Monthly* prints an original poem written in "James Whitcomb Riley English":

I dunno what we're comin' to, it does beat all, to see
What things these doctor fellers do; it's got so now, by gee,
They take a feller's liver'n lights and all his innerds out
And dust 'em off and scour 'em up and put 'em back about
As good as what they ever was. Why, I read t'other day,
They took a woman's stomach out and hove it clean away,
And hicht on some 'f her other works, and now she's up and 'round
And eatin' much as ever, with her stomach under ground!
Now, don't that beat the Dutch? Why, say, b'gosh, I tell yer what!
I thought my stomach was the most important thing I'd got,
And now these fellers chuck 'em 'round as if they didn't mount
To nothin' more'n a worn-out tooth, jest simply no account.
If I'd no stomach I could eat a slice er hot mince pie
And not be groanin' all night long as if I's goin' ter die,
And Jed could chaw green apples, too, and not be doubled up
And keep his ma a-pourin' down pain-killer by the cup;
There'd be no more dyspepsy and the babies wouldn't squall
An hour or two with colic; so, jest take it all in all,
'Twould be a sorter blessin' and I hain't so sure, by jing,
But what I may have mine taken out some time this comin' spring.

---

THE *Daily Chronicle* has from time to time published a series of extremely clever poems in the cockney dialect:

"WHORT FUR?"

"Theer's the doctors: tikes their chawnces when the fever's spreadin' far;
Risk o' life it ain't no bar.
And theer ain't no banners flyin' when they cops it and lies dyin',
Nor no medal nor no star.
When chaps awsts yer whort's the sense?
Whort's the yoose?
Fur why?
Sye the work they did were noble, though it meant they 'ad ter die,
Sort they are
Knows the work they does is wuth it, though it means they 'as ter die."

---

### UNAPPRECIATIVE.

Patient—What would you think of a warmer climate for me, doctor?

Doctor—Good Lord, man! That's what I'm trying to save you from.

